# Influence of Particle Size on Compressive Strength of Alkali Activated Refractory Materials

**DOI:** 10.3390/ma13102227

**Published:** 2020-05-12

**Authors:** Barbara Horvat, Vilma Ducman

**Affiliations:** Slovenian National Building and Civil Engineering Institute, Dimičeva ulica 2, 1000 Ljubljana, Slovenia

**Keywords:** refractory materials, alkali activation, particle size, SEM, EDXS, XRF, XRD, compressive strength

## Abstract

Influence of particle size on the mechanical strength of alkali activated material from waste refractory monolithic was investigated in this study. Precursor was chemically and mineralogically analysed, separated on 4 fractions and alkali activated with Na-water glass. Alkali activated materials were thoroughly investigated under SEM and XRD to evaluate the not predicted differences in mechanical strength. Influence of curing temperature and time dependence at curing temperatures on mechanical strength were investigated in the sample prepared from a fraction that caused the highest compressive strength.

## 1. Introduction

Alkali activated materials (AAM) are prepared from precursors that are rich in amorphous Si and Al. When the precursor and liquid alkali are mixed, dissolution of ingredients takes place, followed by diffusion and rearranging of chemical elements into monomer and finally into polymer aluminosilicate network (ASN) made from SiO_4_ and AlO_4_ tetrahedrons that are linked by O-bridges. Negative charge of Al in the ASN is compensated with positive ions from the 1st group of the periodic system from the alkali and with positive ions present in the precursor that get dissolved in the liquid mixture [1]. AAM represents potential future replacement of nowadays products for and from building industry, i.e., for cement, mortar, concrete, bricks, fire-resistant panels, pavements, ceramics [2,3,4,5,6,7,8], etc. The cost of these products is lowered due to using waste material instead of raw materials, i.e., no excavating of raw materials, no need for dumping waste in the municipal dumps, no need for downcycling and recycling because waste material becomes new raw material which is then upcycled with less need for energy and in a shorter time than conventional products [9,10,11].

When using waste material as the precursor, the material itself can contain ingredients that react with aqueous alkali with gas release, resulting in alkali activated self-foam (AAsF). This reaction can take place instantly or has a delayed effect (AAdF).

Influence of particle size on compressive strength of AAM was published in several papers stating that smaller the precursor’s particles, higher the compressive strength [12,13,14,15,16,17,18,19]. This effect was also investigated in connection to the synthesis of alkali activated foam, where the same dependence was found, i.e., smaller the particles, higher the compressive strength [20,21]. In addition with small enough particles in the slurry, slurry goes from Bingham plastic to Newtonian liquid, viscosity decreases and larger pores form (easier escape of the gasses from the slurry).

In this paper, refractory monolithic was investigated regarding their suitability for alkali activation, and specific focus has been put on the influence of particle size on the mechanical properties of AAM. Monolitics are materials that have no definite shape, can be used on-site for repair or construction of furnaces; consist of aggregates, binders and fillers [22].

## 2. Materials and Methods

### 2.1. Analysis of Precursor and Its Alkali Activated Counterpart

Refractory material (RM), labelled 16 11 06, i.e., waste refractory monolithics for the steel, iron, cement and heat industries, was collected in a manner to be representative of waste dump pile at company Termit, where 1500 t of this waste is collected per year.

Dried (70 °C for 24 h in heating chamber WTB Binder), ground (with the vibrating disk mill Siebtechnik TS250, Siebtechnik GmbH, Mülheim an der Ruhr, Germany) and sieved (below 125 µm) precursor was melted into discs (precursor was mixed with Fluxana_(s)_ FX-X50-2, Fluxana GmbH & Co. KG, Bedburg-Hace, Germany, i.e., a mixture of Lithium tetraborate and Lithium metaborate (mass ratio 1:1), to lower the melting point, in mass ratio 1:10 for Fluxana; LiBr_(l)_ (prepared from 50 mL H_2_O and 7.5 g of LiBr_(s)_ from Acros Organics) was added to the mixture of precursor and Fluxana to avoid gluing of melt onto platinum vessel). On discs, X-ray fluorescence was performed (XRF; Thermo Scientific ARL Perform’X Sequential XRF, Thermo Fisher Scientific Inc., Walthem, MA, USA), measured data were analysed with software UniQuant 5 [version 5.00, Thermo Fisher Scientific Inc., Walthem, MA, USA].

X-ray powder diffraction (XRD; Empyrean PANalytical X-ray Diffractometer, Malvern PANalytical Empyrean, Netherlands and United Kingdom, Cu X-Ray source) was performed on the dried, ground and sieved (below 125 µm) precursor and alkali activated counterpart. XRD pattern was solved with software X’Pert Highscore plus 4.1 (4.1, Malvern PANalytical, Malvern, UK). Amount of amorphous phase and minerals were estimated with Rietveld refinement by using an external standard (pure corundum, Al_2_O_3_).

Surface, shape and microstructure investigation of un-sputtered dried (at 40 °C in vacuum for 24 h in Kambič heating chamber, VS 50 S) precursor and alkali activated materials was performed with the scanning electron microscope (SEM; Jeol JSM-IT500, Jeol, Tokyo, Japan) under high vacuum conditions. Energy-dispersive X-ray spectroscopy (EDXS; Oxford Instruments, Link Pentafet, London, UK) mapping of polished un-sputtered alkali activated samples were performed in low vacuum conditions.

Bending and compressive strength of alkali activated materials were measured with compressive and bending strength testing machine (ToniTechnik ToniNORM, Toni Technik, Berlin, Germany) 1 week after demoulding.

By weighting the alkali activated prisms and dividing its mass with its volume the geometrical densities of alkali activated materials were determined.

### 2.2. Preparation of Precursors, Activator and Alkali Activated Samples

**Figure 1:**
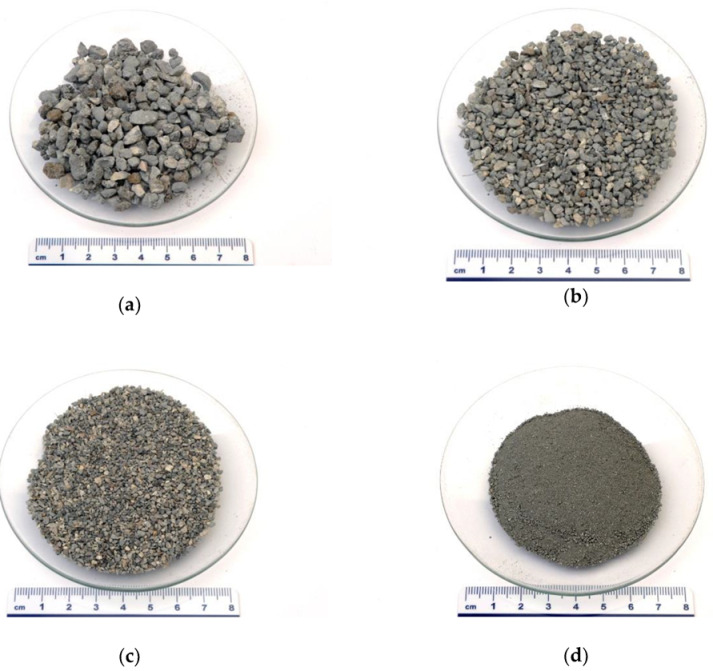
Photographs of refractory material (RM) sieved (**a**) above 4 mm, (**b**) between 2 mm and 4 mm, (**c**) between 1 mm and 2 mm and (**d**) below 1 mm.

The precursor used for alkali activation was dried at 70 °C for 24 h and sieved afterwards into 4 fractions: above 4 mm (4 mm < x), between 2 mm and 4 mm (2 mm < x ≤ 4 mm), between 2 mm and 1 mm (1 mm < x ≤ 2 mm) and below 1 mm (x ≤ 1 mm), all shown in Figure 1. On all fractions of precursors, XRF and XRD measurements were performed in the manner described in Section 2.1. Fractions were alkali activated with Na-water glass (Geosil, 344/7, Woelner, with the mass percentage of Na_2_O 16.9 %, and mass percentage of SiO_2_ 27.5 %) in mass ratio 50:30 for precursor (lower amount of alkali glass was not enough for wetting). Slurries were put into PVC moulds of size (80 × 20 × 20) mm^3^ and cured in heating chamber WTB Binder at 70 °C for 24 h. Prisms were demoulded after cooling down to room temperature and left at room temperature for an additional 7 days.

Alkali activated sample prepared from the fraction that had the highest compressive strength was additionally synthesized and cured at room conditions, at 40 °C and 70 °C for 7 days. Compressive strength was tested at days 7 and 28.

## 3. Results and Discussion

### 3.1. Analysis of Precursor

**Figure 2:**
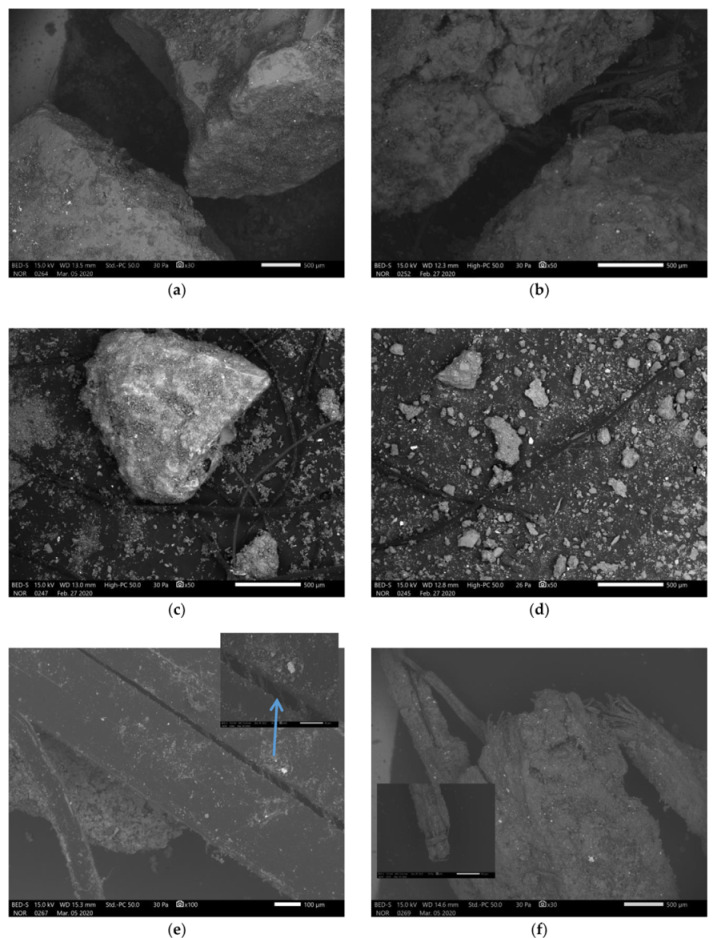
SEM micrographs of RM sieved (**a**,**e**,**f**) above 4 mm, (**b**) between 2 mm and 4 mm, (**c**) between 1 mm and 2 mm and (**d**) below 1 mm.

SEM micrographs of precursor prepared to be used in alkali activation are presented in Figure 2. All fractions contain polymer fibres and all contain small particles that are present on the surface of the bigger particles, on the fibres, aggregated as the fibre “glue” and are present as “dust” in all fractions (bigger fractions were not washed off fine powder). Particles’ shape is random with not a smooth surface and less sharp edges. Fibres in the biggest fraction are presented in Figure 2e, large single fibre splitting into smaller fibres and not completely split in the middle, with precursor on the surface, and in Figure 2f, a bundle of fibres completely wrapped in the precursor.

**Figure 3:**
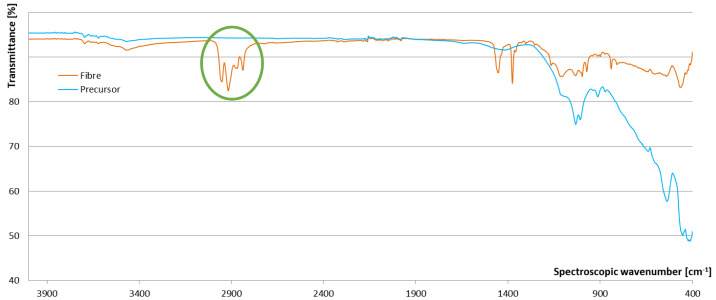
FTIR measurement of precursor sieved below 1 mm (blue line), and fibre (orange line).

FTIR results of precursor sieved below 1 mm (without visible fibres) and of fibre without visible precursor on its surface, are presented in Figure 3. According to the literature [23] fibres are polyvinyl chloride (PVC; characteristic peaks are in the orange circle), which are resistant to diluted alkali [24].

Chemical analysis of all precursor’s fractions is presented in Table 1, where in 1st rows are mass percentages of elements crucial for alkali activation measured with XRF, in 2nd rows, mass percentages of elements crucial for alkali activation in crystalline phase measured with XRD and determined by means Rietveld refinement (GOF for all analysis around 8 (waste material)), in 3rd rows the mass percentage difference between the mass percentage of all elements crucial for alkali activation and mass percentage of elements crucial for alkali activation in the crystalline phase, i.e., the amount of amorphous phase. In all fractions, there is a small amount of elements from 1st and 2nd group of the periodic system, all except Ca are in the amorphous phase. Amorphous part of Ca represents the highest value among them, i.e., in all fractions more than 1 mass % (m_%_). As a good potential precursor for alkali activation, it does contain a decent amount of Al and Si, both in the crystalline and amorphous phase. However, there is (in all fractions) more Al than Si meaning that according to the literature [25] material has the tendency to foam, as there is always Si:Al ≤ 1.40 (0.70, 0.81, 0.82, 0.86 from biggest to smallest fraction if all amorphous material is accessible in the reaction and if minerals do not dissolve in alkali).

**Figure 4:**
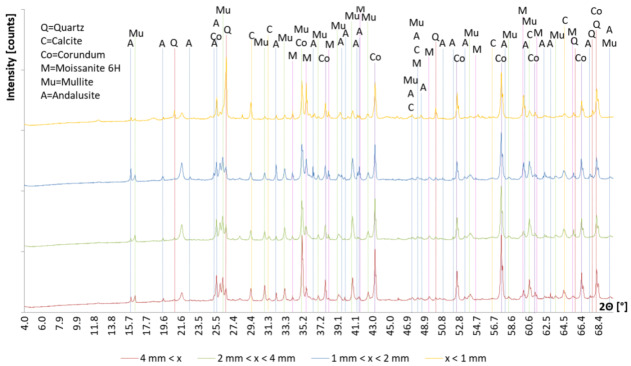
XRD pattern of precursor’s fraction above 4 mm (red line), between 2 mm and 4 mm (green line), between 1 mm and 2 mm (blue line) and below 1 mm (orange line). In the legend, minerals are: quartz (SiO_2_, card number ICSD 98-015-6196), calcite (CaCO_3_, card number ICSD 98-015-8258), corundum (Al_2_O_3_, card number ICSD 98-016-0604), moissanite 6H (SiC, card number ICSD 98-015-6190), mullite (Al_1.83_O_4.85_Si_1.08_, card number ICSD 98-004-3298) and alusite (Al_2_SiO_5_, card number ICSD 98-008-4614).

**Figure 5:**
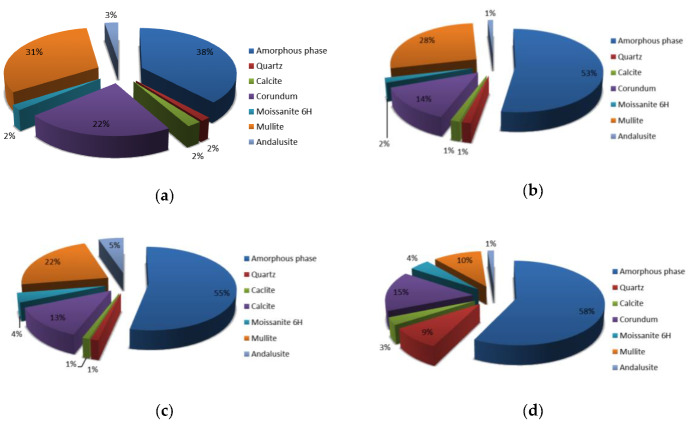
Rietveld refinement of precursor’s fraction (**a**) above 4 mm, (**b**) between 2 mm and 4 mm, (**c**) between 1 mm and 2 mm and (**d**) below 1 mm.

In Figure 4 are presented the XRD analysis of all precursor’s fractions and in Figure 5 their Rietveld refinement results. Amount of amorphous phase increases with smaller fraction of the precursor, also the amount of mullite. Amount of moissanite, calcite and andalusite is in all fractions similar, while the amount of quartz is highest in the smallest precursor’s fraction (other fractions have a comparable amount of quartz), and amount of corundum is biggest in the largest precursor’s fraction (other fractions have a comparable amount of corundum). On XRD patterns presented in Figure 4 is a peak (between 21° and 22°) present in all fractions which could not have been determined.

### 3.2. Analysis of Alkali Activated Samples Prepared from Different Precursor’s Fractions

Compressive and bending strength of all alkali activated samples prepared from different fractions of the precursor are presented in Table 2 along with the density of the final products. Density and mechanical strengths grow from largest to 2nd smallest precursor (particles from 1 mm to 2 mm, blue row in Table 2), which is in agreement with the literature [16], but declines drastically when precursor’s fraction gets below 1 mm, which contradicts literature [16]. The 2nd smallest precursor’s fraction had from more than 3-times higher compressive strength from the smallest precursor’s fraction, while density declined only for just more than 1/3, indicating that self-foaming (chemical reaction with alkali) played a bigger role in the alkali activated synthesis, i.e., more sample reacted (due to bigger available precursor’s surface) and there was not enough free space between not yet reacted inner part of the precursor’s particles to allow gasses to escape without being trapped (creating pores) in the structure. Self-foaming was observed in alkali activation of the precursor with particle’s fraction below 1 mm, which is the reason for lower compressive strength in comparison to the alkali activated material synthesized from bigger fractions.

**Figure 6:**
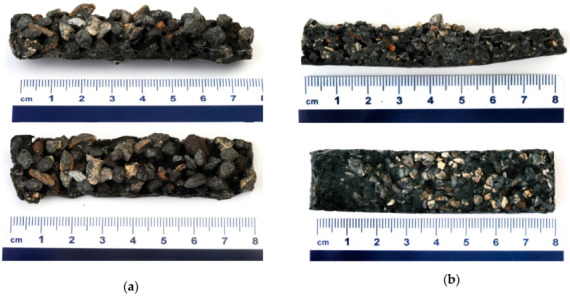

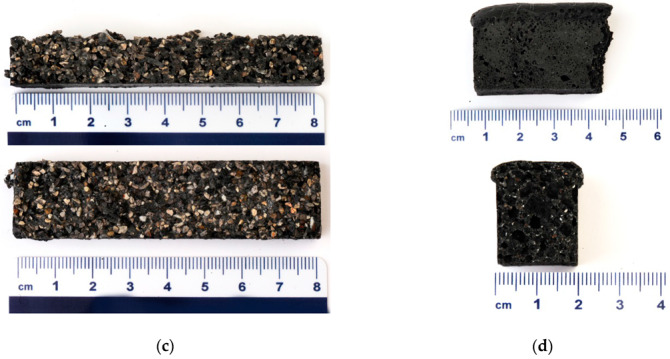
Photographs of RM sieved (**a**) above 4 mm, (**b**) between 2 mm and 4 mm, (**c**) between 1 mm and 2 mm and (**d**) below 1 mm.

In Figure 6 are presented photographs of all alkali activated refractory material fraction’s where the largest 3 fractions are bonded together in the ASN, while the smallest fraction (Figure 5d) has a completely different structure, i.e., porous and almost completely reacted.

**Figure 7:**
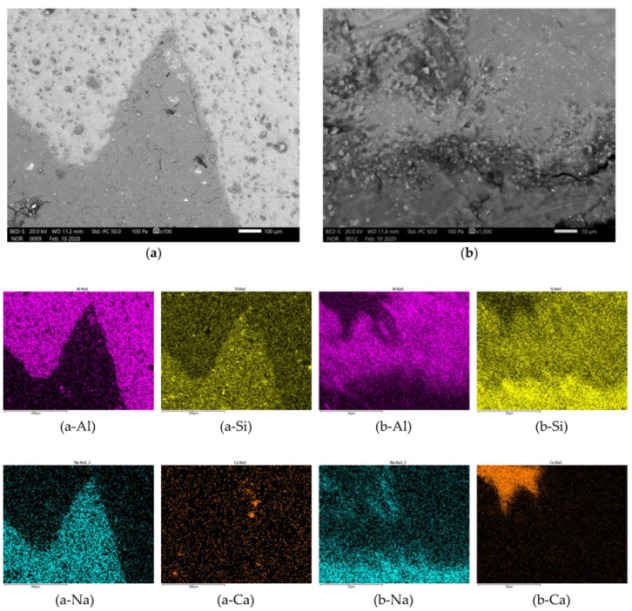
SEM micrograph of alkali activated material prepared from precursor’s fraction 4 mm < x with magnification of (**a**) 100 and (**b**) 1000. EDXS mapping is presented in (a/b-chemical element) of area (a/b), respectively.

**Figure 8:**
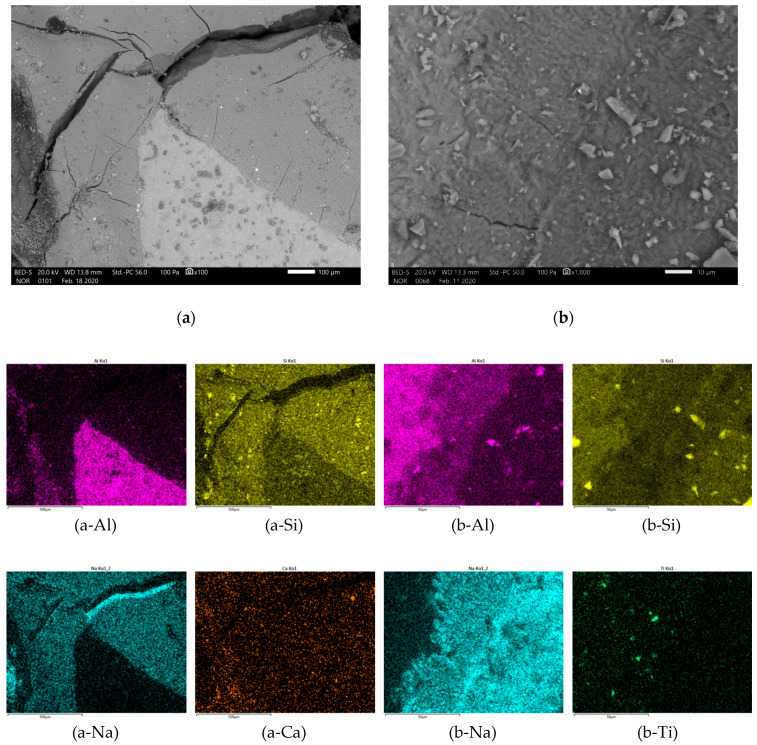
SEM micrograph of alkali activated material prepared from precursor’s fraction 2 mm < x ≤ 4 mm with magnification of (**a**) 100 and (**b**) 1000. EDXS mapping is presented in (b-chemical element) of area (b).

**Figure 9:**
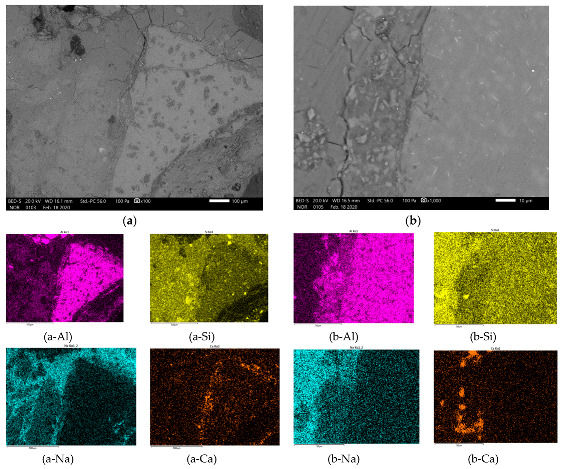
SEM micrograph of alkali activated material prepared from precursor’s fraction 1 mm < x ≤ 2 mm with magnification of (**a**) 100 and (**b**) 1000. EDXS mapping is presented in (b-chemical element) of area (b).

**Figure 10:**
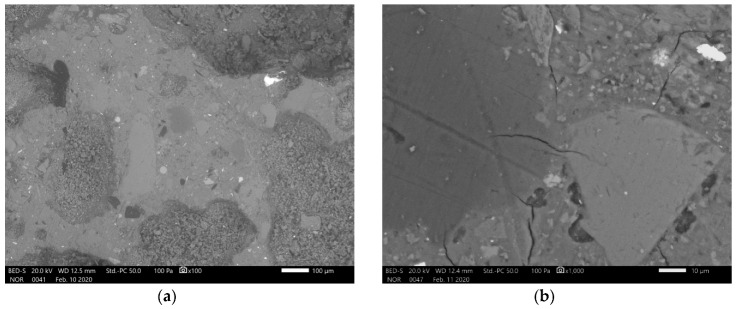

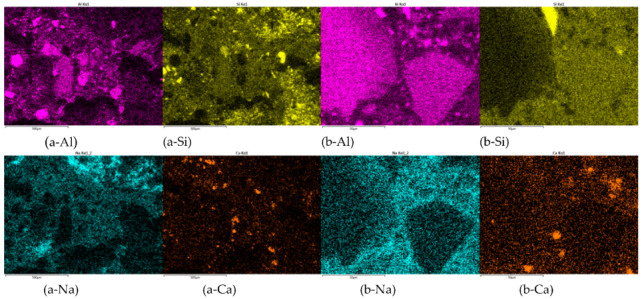
SEM micrograph of alkali activated material prepared from precursor’s fraction x ≤ 1 mm with magnification of (**a**) 100 and (**b**) 1000. EDXS mapping is presented in (b-chemical element) of area (b).

In Figure 7, Figure 8, Figure 9 and Figure 10 is presented polished unsputtered cross-sections of alkali activated material prepared from precursor’s fraction above 4 mm (4 mm < x), between 2 and 4 mm (2 mm < x ≤ 4 mm), 1 and 2 mm (1 < x ≤ 2 mm) and below 1 mm (x ≤ 1 mm), respectively. As it is obvious in Figure 6 that alkali activation of all fractions above 1 mm particle’s size resulted in AAM consisting of non-reacted precursor’s particles and “binder” connecting them (large Al particles did not dissolve; therefore reaction could take place only on the surface of particles), also SEM micrographs (Figure 7, Figure 8 and Figure 9) show comparable results: darker grey part presents ASN consisting mostly of Si and Na, while white parts present not-dissolved Al. Cracks (lower the mechanical strength) are present only in ASN due to curing and chemical reactions throughout the whole ASN independently on the presence of the not-reacted precursor’s particles: cracks can be perpendicular, longitudinal on the border ASN and not-reacted precursor, or the border can be chemically “erased” due to the chemical reaction binding ASN and not-reacted precursor, which is presented on Figure 8b and its EDXS analysis.

AAM from smallest precursor’s fraction (Figure 10) has visible cracks, present in ASN, only under higher magnification, but has large pores (lower the density and mechanical strength of AAM) visible under low magnification. ASN contains also not-reacted precursor’s particles which are spread throughout the ASN and are well incorporated in it. Smaller and fewer cracks present in the ASN in comparison to AAM from larger precursor’s fractions are a consequence of gasses getting trapped in the slurry and later in the hardened ASN, giving pressure onto the ASN structure also from the inside and not creating additional cracks through which gasses and liquid escaped while curing/drying took place.

With control of the particle’s size of precursor, it is possible also to control packing of the particles, i.e., the smaller the particles the closer the packing, which results in higher mechanical strength (experiment from precursor’s particles between 1 mm and 2 mm). With particles’ size, it is also possible to control the size of the voids which allow the escape of gasses or get filled with dissolved material in the slurry from which ASN forms.

**Figure 11:**
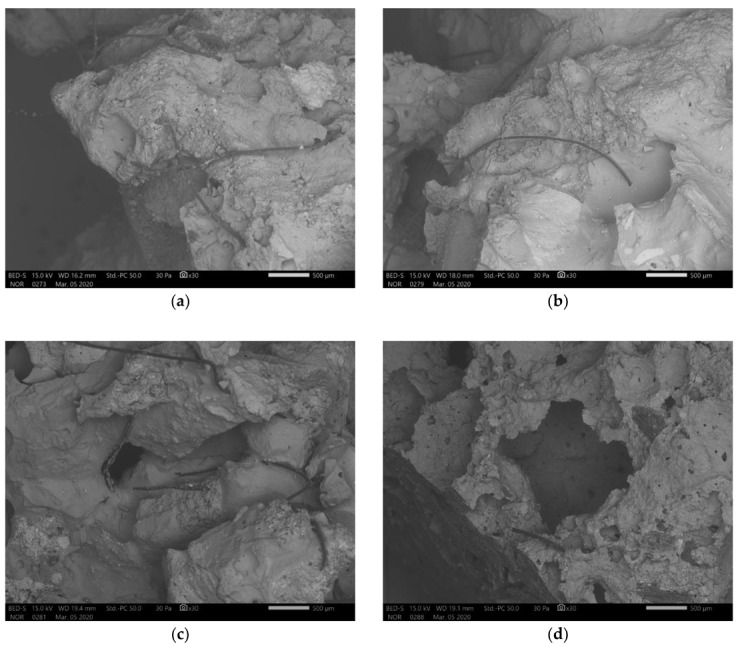
SEM micrographs of unpolished alkali activated materials (AAM) after bending strength measurement from precursor sieved (**a**) above 4 mm, (**b**) between 2 mm and 4 mm, (**c**) between 1 mm and 2 mm and (**d**) below 1 mm.

In Figure 11 are presented SEM micrographs of unpolished AAM after bending strength measurement from all precursor’s fractions. Artificial fibres did not react with alkali, but due to the small precursor’s particles being present on its surface, it got well incorporated into the AAM. It is predicted that fibres offer increased bending strength, but since they cannot be completely removed (are present in all fractions) an experiment without fibres is not possible.

**Figure 12:**
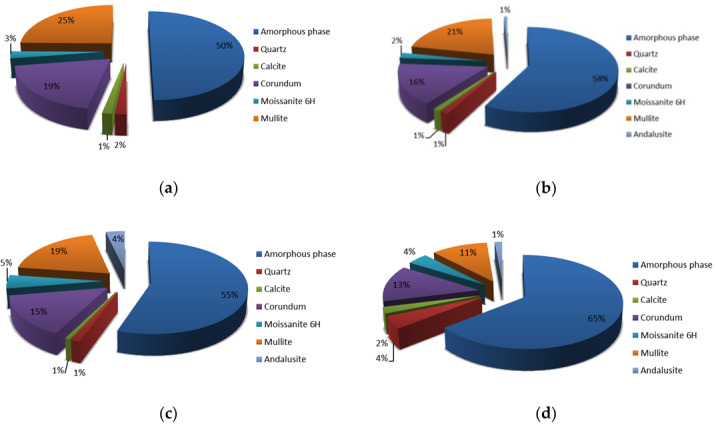
Rietveld refinement of alkali activated materials from fraction of precursor (**a**) above 4 mm, (**b**) between 2 mm and 4 mm, (**c**) between 1 mm and 2 mm and (**d**) below 1 mm.

In Figure 12 are presented Rietveld refinement (GOF around 7 for all analysis) results for AAM from all fractions. Comparison of amounts of amorphous phase and minerals present in AAM from different fractions shows similar dependence to amounts present in the precursor’s fractions.

### 3.3. Analysis of Alkali Activated Sample Cured at Different Temperatures

Highest compressive and bending strength among all alkali activated samples prepared from different fractions had the sample that was prepared from precursor’s fraction between 1 mm and 2 mm (see Table 2). This fraction was alkali activated again in the same manner, just curing was performed on different temperatures (room conditions, 40 °C and 70 °C) for longer time (7 days instead of 1 day). Mechanical strengths of these samples measured at day 7 and 28 are presented in Table 3. Alkali activated material cured at room temperature did not gain measurable mechanical strengths at 7 days (it was not solidified yet), but it gained it at 28 days when both strengths were still much lower comparing to the samples treated at higher curing temperatures. From the Table 3, it is also obvious that the higher the temperature, higher the compressive and bending strengths at 7 and 28 days and the longer the time after moulding, the higher the mechanical strengths [26]. According to the ratio of the increase of mechanical strength over the time, we can conclude, that mechanical strengths are near the maximal value, while samples cured at lower temperatures still need much more time to reach the limit value. When comparing sample cured at 70 °C for 1 day and left at room conditions for an additional 6 days (Table 2), and cured at 70 °C for 7 days (Table 3), sample cured on a higher temperature for a longer time reached 80 % of compressive strength of sample cured on the same temperature for a shorter time.

### 3.4. Proposed Mechanism behind AAM Structure from Different Precursor’s Fractions

From photos and SEM micrographs of AAM, prepared from different precursor’s fractions, presented in Section 3.2., it is obvious that self-foaming effect showed high impact only in the fraction below 1 mm, i.e., in the smallest fraction. Diffusion of dissolved precursor’s compounds and their distribution have an important impact in “polymerization” in alkali activated synthesis, and should be limited to the local surroundings (diffusion limited “aggregation”) to gain faster solidification (property desired in the industry). This can be gained with the addition of the lowest amount of liquid possible to the precursor, i.e., to have highest possible viscosity of the slurry (drying needs less time and diffusion is limited with high viscosity) [26], that still offers in this short reactive time a sufficient bonding in the matrix. The unreacted part of particles serves as an aggregate which is chemically bonded with the matrix to certain degree depending on diffusion of all reactive elements in the slurry.

**Figure 13:**
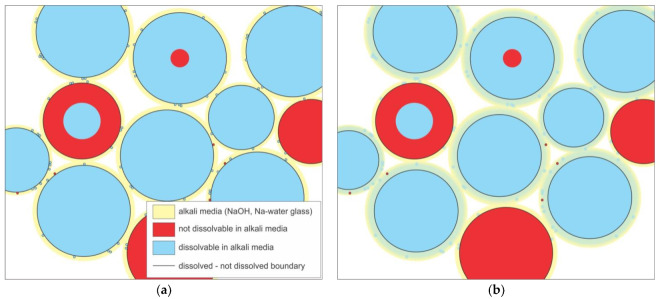

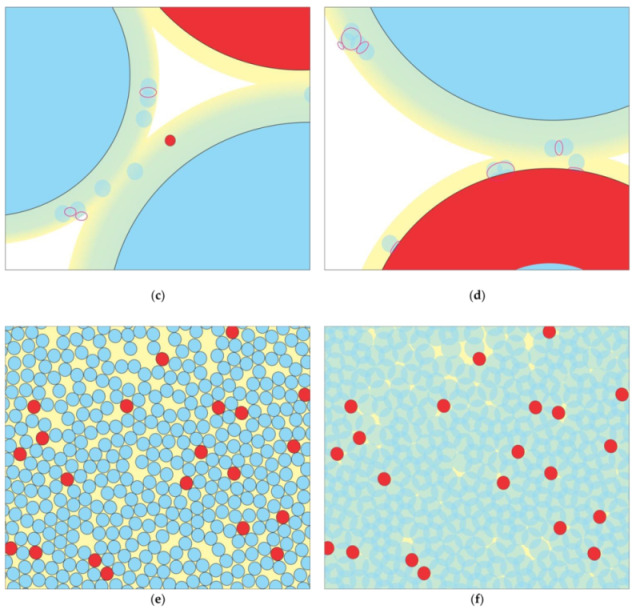
Sketches of early alkali activation process from big (**a**–**d**) and small (**e**,**f**) precursor. The 1st step (**a**,**e**) is wetting of the precursor’s surface, 2nd step (**c**,**d**,**f**) is the dissolution of in alkali dissolvable precursor’s ingredients accompanied by diffusion of dissolved ions.

In Figure 13 are sketches of large (a–d) and small (e,f) fractions of the precursor from in alkali (yellow), dissolvable (blue) and not dissolvable (red) compounds. After wetting of the surface (Figure 13a,e) with alkali, blue compounds start to dissolve (to the black line marking the point to which dissolution is reached) and diffuse in the media (Figure 13b,f). With diffusion, the amount of Al and Si changes locally (seen on EDXS mapping of Al represented in Figure 8b-Al and Figure 9b-Al).

Gasses produced in the foaming reaction can be released into the large vacancies in the packing of the partially dissolved particles of precursors (seen also on photographs in Figure 6a–c). The foaming mechanisms which has not been definitely confirmed might be a consequence of partial dissolution of SiC (present in mineral Moissanite 6H) in alkali media [27] react with alkalis and oxygen and forms gas CO_2_. These gas(es) then shape the material from within with bubbles transforming AAM into alkali activated (self-)foam (AAsF).

## 4. Conclusions

Compressive and bending strength of alkali activated material showed unique dependence on refractory material’s particle’s fraction where down to certain fraction (down to 1 mm) mechanical strength is increasing, i.e., the smaller the particles, the bigger the reactive surface area in comparison to the volume, the higher reaction rate of the precursor and more reacted final material, which would make AAM with better mechanical strength if alkali activation would not cause precursor to foam, i.e., when alkali activating precursor has tendency to self-foam, the mechanical strength depends on the amount of reacted material and porous structure in the reacted material. If gases can be released from the AAM through the voids between particles in a larger fraction of the precursor, they do not affect the mechanical strength as much as when they destroy the compactness of the AAM leaving it with lower density, and lower compressive and bending strength. Highest mechanical strength had a sample with fraction 1 mm < x < 2 mm, which had the best close packing of not-reacted precursor’s particles and still was not influenced by foaming effect. Mechanical strength of AAM shows dependence on curing temperature and time after AAM synthesis. The higher the temperature and the longer the time after synthesis, the higher the mechanical strength.

## Figures and Tables

**Table 1:** 1st row of precursor’s fractions presents the mass percentage of elements measured with X-ray fluorescence (XRF), 2nd row mass percentage of elements in the crystalline phase that was determined with Rietveld refinement from XRD, 3rd row mass percentage of elements in the amorphous phase.

Precursor’s Fraction	Elements [m_%_]	Na	K	Cs	Mg	Ca	Sr	Ba	Al	Si
4 mm < x	XRF	0.20	0.21	0	0.62	2.19	0.04	0.20	32.71	12.61
	XRD	0	0	0	0	0.88	0	0	22.59	5.56
	XRF-XRD	0.20	0.21	0	0.62	1.31	0.04	0.20	10.12	7.05
2 mm < x ≤ 4 mm	XRF	0.25	0.27	0	0.45	1.69	0.03	0.10	30.46	15.27
	XRD	0	0	0	0	0.60	0	0	18.44	5.50
	XRF-XRD	0.25	0.27	0	0.45	1.09	0.03	0.10	12.02	9.77
1 mm < x ≤ 2 mm	XRF	0.24	0.29	0	0.40	2.04	0.03	0.13	30.05	16.83
	XRD	0	0	0	0	0.44	0	0	17.56	6.56
	XRF-XRD	0.24	0.29	0	0.40	1.60	0.03	0.13	12.49	10.27
x ≤ 1 mm	XRF	0.48	0.24	0	0.46	2.63	0.03	0.22	25.79	20.01
	XRD	0	0	0	0	1.20	0	0	12.36	8.42
	XRF-XRD	0.48	0.24	0	0.46	1.43	0.03	0.22	13.43	11.59

**Table 2:** The density of alkali activated samples, their compressive and bending strengths in dependence of precursor’s particles’ fraction.

Precursor’s Fraction	Compressive Strength [MPa]	Bending Strength [MPa]	Density [kg/L]
4 mm < x	3.9	2.2	1.2
2 mm < x ≤ 4 mm	14.1	5.3	1.3
1 mm < x ≤ 2 mm	21.3	8.6	1.5
x ≤ 1 mm	6.4	3.5	0.9

**Table 3:** The compressive and bending strengths of alkali activated samples from fraction 1 mm to 2 mm, in dependence of curing temperature and elapsed time.

Curing Temperature for 7 Days	Compressive Strength after 7 Days [MPa]	Compressive Strength after 28 Days [MPa]	Bending Strength after 7 Days [MPa]	Bending Strength after 28 Days [MPa]
Room conditions	0	1.8	0	1.9
40 °C	2.4	4.1	0.05	3.6
70 °C	17.4	20.4	7.6	9.0
